# Zhenbao Pill reduces Treg cell proportion in acute spinal cord injury rats by regulating TUG1/*miR-214*/HSP27 axis

**DOI:** 10.1042/BSR20180895

**Published:** 2018-11-07

**Authors:** Yongxiong He, Mingdong Li, Wujisiguleng, Bokang Lv, Yanqiang Huan, Bin Liu, Dongsheng Wang, Hai Yu, Liansheng Zhang, Zhiqiang Shi

**Affiliations:** 1 Department of Spine Surgery, Inner Mongolia People’s Hospital, Hohhot 010017, Inner Mongolia, China; 2 Department of Orthopaedic and Traumatology, Hainan General Hospital, Haikou 570311, Hainan, China; 3 Department of Orthopedic Surgery, Affiliated People’s Hospital of Inner Mongolia Medical University, Hohhot 010020, Inner Mongolia, China; 4 Department of Emergency Surgery, The Second Affiliated Hospital of Inner Mongolia Medical University, Hohhot 010030, Inner Mongolia, China

**Keywords:** Foxp3, miR-214, SCI, TUG1, Treg cell, Zhenbao Pill

## Abstract

**Background:** Acute spinal cord injury (SCI) is one of the weakest pathologies that seriously affect the quality of life of patients. **Objective:** To study the mechanism of how Zhenbao Pill reduces Treg cell proportion and improves acute SCI. **Methods:** A rat SCI model was established. Flow cytometry analysis was performed to determine the Treg cell proportion. RNA immunoprecipitation (RIP) and RNA pull-down were applied in confirming taurine up-regulated gene 1 (TUG1) and *miR-214* binding. Intrathecal injection of TUG1 siRNA was also conducted to determine the effect of TUG1 *in vivo*. **Results:** Zhenbao Pill promoted the expression of TUG1 and heat shock protein 27 (HSP27) protein, and reduced the expression of *miR-214* and forkhead box protein p3 (Foxp3) as well as Treg cell proportion in a concentration-dependent manner in SCI rats or *in vitro* cultured CD4^+^ T cells. Knockdown of TUG1 reversed the high protein expression of HSP27 and the inhibition of Treg cell proportion as well as Foxp3 protein induced by Zhenbao Pill, and *miR-214* inhibitor canceled the TUG1 knockdown effect. Further, *miR-214* mimic reversed the inhibition of Treg cell proportion and Foxp3 protein expression by Zhenbao Pill, which was abolished by the overexpression of HSP27. The mechanism was validated in animal experiments. **Conclusion:** Zhenbao Pill regulated TUG1/*miR-214*/HSP27 signaling pathway to reduce Treg cell proportion and thus relieve acute SCI.

## Introduction

Spinal cord injury (SCI) can be divided into primary injury and secondary injury [1,2]. Primary injury mainly refers to the destruction of the spinal cord structure caused by external forces. Secondary injury is mainly caused by primary injury, and the main cause of SCI is the sustained damage caused by inflammation of injured spinal cord tissue, immune damage, and cell apoptosis [3,4]. Major damage to spinal cord function and structure is irreversible. Therefore, the study of ASCI treatment and rehabilitation mainly focussed on the secondary injury [5–7].

Zhenbao Pill consists of 29 Chinese herbal medicines, including pearl, cassia seed, bezoar, saffron, amomum, and licorice, which promote blood circulation, activate meridians, and calm nerves. Clinically, Zhenbao Pill is used to treat neurological diseases such as stroke and hemiplegia sequelae. Modern pharmacological studies have shown that Zhenbao Pill had the function of repairing damaged neurones, promoting microcirculation, and removing oxygen free radicals [8,9]. Our previous research has found that Zhenbao Pill had a protective effect on the nerves of SCI rats [10]. We also found that Zhenbao Pill improved the performance of SCI rats in the Basso, Beattie, and Bresnahan (BBB) score and reduced the proportion of Treg cells [11].

Currently, it has been confirmed that the reduction in Treg cells and thus the alleviation of systemic immunosuppression can promote the repair of spinal cord injuries [12]. Our previous study shown that Zhenbao Pill inhibited the Treg lymphocyte numbers and the decrease in TGF-β levels by inhibiting the *miR-214* induced expression of heat shock protein 27 (HSP27) during the repair of SCI [11]. However, studies are still required to further elucidate the underlying molecular mechanism of how Zhenbao Pill reduces the proportion of Treg cells and improves ASCI.

Based on software (LncBase Predicted v.2) prediction, lncRNA taurine up-regulated gene 1 (TUG1) can bind to *miR-214*. TUG1 has been considered as an oncogene in many tumor cells [13,14]. Recent studies have demonstrated that TUG1 was expressed in peripheral blood mononuclear cells (PBMCs) and T lymphocytes [15,16]. However, the role of TUG1 in SCI has not been reported. Therefore, this article aimed to study whether Zhenbao Pill can reduce the proportion of Treg cells by regulating the expression of HSP27 through lncRNA TUG1/*miR-214* and thus improve ASCI.

## Materials and methods

### Animals

Thirty-five young adult female SD rats (180–220 g) were included in the study. Rats were housed in standard conditions (22°C), illumination (12 h light–dark cycle) and constant humidity (55‒65%), having easy access to food and water *ad libitum*, and they were allowed to acclimatize for 1 week before the start of any procedure.

Twenty-one of them were randomly divided into sham group (*n*=7), SCI group (*n*=7), and Zhenbao Pill treatment group (SCI + Zhenbao Pill group, *n*=7). Others (*n*=14) were randomly divided into SCI + Zhenbao Pill + si-control (*n*=7), and SCI + Zhenbao Pill + siRNA-TUG1 (*n*=7). Rats took oral Zhenbao Pill (0.4 g/kg) once daily for 6 weeks based on previous study [17]. The 0.5% sodium carboxymethylcellulose (CMC) reagent was used as the solvent control of Zhenbao Pill. Rats in SCI + Zhenbao Pill + siRNA-TUG1 group received siRNA-TUG1 packaged with lentiviral vector by intraperitoneal injection (5 μl).

### Establishment of SCI model

The animal experiments were performed according to a protocol that was approved by the Animal Ethics Committee of Inner Mongolia People’s Hospital. The establishment of SCI model was conducted as our previous research [18]. In short, rats were anesthetized by the intraperitoneal injection of sodium pentobarbital (40 mg/kg). Then the skin and muscles on the spine were cut and a laminectomy at T9-11 was applied to keep the dura undamaged. The spinal cord was contused using an impactor (2.4 mm diameter, 20 g weight) from the height of 2.5 cm along the guide needle vertical strike T10. Rats in sham group were treated similarly, but without being contused. After surgery, 0.9% physiological saline (30 ml/kg) was injected into rats, avoiding dehydration. Afterward, the rats were housed alone, and assisted urination was performed three times a day. [17]

### Neurological function

Six weeks later, BBB scale was used to assess the neurological function of rats [19]. The BBB scoring criteria was divided into 21 scores. Normal rats have a BBB score of 21, while ASCI rats with completely paralyzed hindlimbs have a BBB score of 0.

### CD4^+^ T-cell isolation and purification

We isolated and purified the CD4^+^ T cells from lymph nodes as previously described [20]. Briefly, the fresh lymph nodes were placed in RPMI medium (containing 2% FBS), and incised by a metal mesh and a plunge. The cell mass and fibrous tissue were then removed by a cell filter. After washing twice, the cells were centrifuged at 300×***g***. Then FITC–conjugated anti-CD4 antibody was used to label the cells by a 10-min incubation at 4°C. The following labeling procedure was conducted using the Anti-FITC Multisort kit (Miltenyi Biotec, Germany). Afterward, the labeled cells were sorted and the CD4^+^ T cells with positive signal can be obtained.

### Quantitative reverse transcription-PCR

Total RNA was extracted with the TRIzol reagent (Invitrogen, Carlsbad, U.S.A.). Then, 2 μg RNA was reverse transcribed into the first-strand cDNA using PrimeScript RT reagent kit (Takara Bio Inc., Shiga, Japan). Quantitative reverse transcription-PCR (qRT-PCR) were performed by using an ABI Prism 5700 Sequence Detection System (Applied Biosystems). All procedures were performed according to the manuals. The relative gene expression was determined with the 2^−ΔΔ*C*t^ approach.

### Western blotting

Equal amounts of protein lysates were loaded on an SDS/polyacrylamide gel (SDS/PAG) for electrophoresis. After transferring on to a PVDF membrane, the proteins were probed with primary antibodies at 4°C overnight. Then the horseradish peroxidase (HRP)-labeled secondary antibody was used. Bands were detected by chemiluminescence (ECL). The used primary antibodies were listed as follows: HSP27 (1:1000, Abcam, Shanghai), forkhead box protein p3 (Foxp3) (1:1000, Abcam, Shanghai), protein argonaute 2 (AGO2) (1:1000, Cell Signaling Technology), and β-actin (1:1000, Abcam, Shanghai). β-actin was used as a loading control.

### Treg cell differentiation induction

The 24-well cell culture plates were coated overnight with filter-sterilized anti-CD3 solution (5 μg/ml in PBS, 200 μl/well). After removing the coating solution, purified CD4^+^ naive cells (2.5 × 10^5^ cells/well) were seeded. Then the anti-CD28 (2 μg/ml) and TGF-β1 (2 ng/ml) solutions were added, and the cells were cultured for 72 h [20].

### Flow cytometry

Flow cytometry analysis was performed to determine the relative amount of Treg cells as described earlier [20]. Briefly, the cells were first stained by the anti-CD4 (1:100, Abcam, Shanghai) and anti-CD25 (1:100, Abcam, Shanghai) antibodies. Subsequently, the cells were fixed as well as permeabilized using the fixation/permeabilization solution kit (BD Cytofix/Cytoperm, U.S.A.) followed by being stained with the anti-Foxp3 antibody (1:50, Cell Signaling Technology). Samples were sorted on apparatus and the relative amount of Treg cells was obtained. Treg cells were measured by flow cytometer with Foxp3+ as the marker.

### RNA immunoprecipitation

Co-immunoprecipitation of AGO2-bound RNAs was conducted as reported earlier [21]. In brief, the 293T cells were first cross-linked with 0.3% formaldehyde. Approximately 1.0 × 10^7^ cells were collected and lysed. Then, the protein A/G magnetic beads (Cell Signaling Technology) coupled with AGO2 (Cell Signaling Technology) or IgG control antibody were added into the cell lysates, and incubated at 4°C overnight. After extensive washing, AGO2-bound RNAs were eluted using elution buffer containing proteinase K, followed by reverse cross-linking. The eluted RNA was purified and reverse transcribed to cDNA according to the manuals.

### RNA pull-down

The biotinylated DNA probe for lncRNA TUG1 was produced by Cell Signaling Technology. The streptavidin-magnetic beads (Invitrogen) were blocked by RNase-free BSA and yeast tRNA, and incubated with biotinylated DNA probe for 2 h in binding buffer. Afterward, the 293T cell lysates were incubated with the pretreated beads for 1 h at 4°C. After extensive washing, the RNA complex was eluted and used for qRT-PCR. AGO2 was examined by Western blotting.

### Cell transfection

The 293T cells were transfected with TUG1 overexpression vector (pcDNA-TUG1), TUG1 siRNA (siRNA-TUG1) or corresponding negative control using Lipofectamine 2000 (Invitrogen) according to the instructions of the manufacturer.

### Lentivirus transfection

The siRNA-TUG1, TUG1, *miR-214* mimic, *miR-214* inhibitor, siRNA-HSP27, or HSP27 was constructed into the lentiviral vector [22]. The lentivirus was packaged and amplified in HEK293T cells, and then used to infect the CD4^+^ T cells at a multiplicity of infection (MOI) of 5. Twenty-four hours later, the transfection efficiency could be determined by qRT-PCR.

### Intrathecal injection of siRNA-TUG1

The intrathecal injection was conducted immediately following SCI [23]. Briefly, the SCI rats were randomly divided into two groups, Zhenbao Pill + si-control group and Zhenbao Pill + siRNA-TUG1 group. siRNA-TUG1 or its negative control (20 nmol/ml) was injected into the intrathecal space of rat ASCI models. All rats received the intramuscular injection of penicillin G (40000 U) in quadriceps femoris during operation, which was continued once daily for 5 days postoperatively.

### Statistical analysis

SPSS version 19.0 statistical software was used for analysis. All data were shown as mean ± S.D. Student’s *t* test or ANOVA was used to analyze the differences between two groups or more than two groups, respectively. *P*<0.05 was taken as statistically significant.

### Ethics approval and consent to participate

The animal experiments were performed according to a protocol that was approved by the Animal Ethics Committee of Inner Mongolia People’s Hospital.

## Results

### The effects of Zhenbao Pill in SCI rats

**Figure 1:**
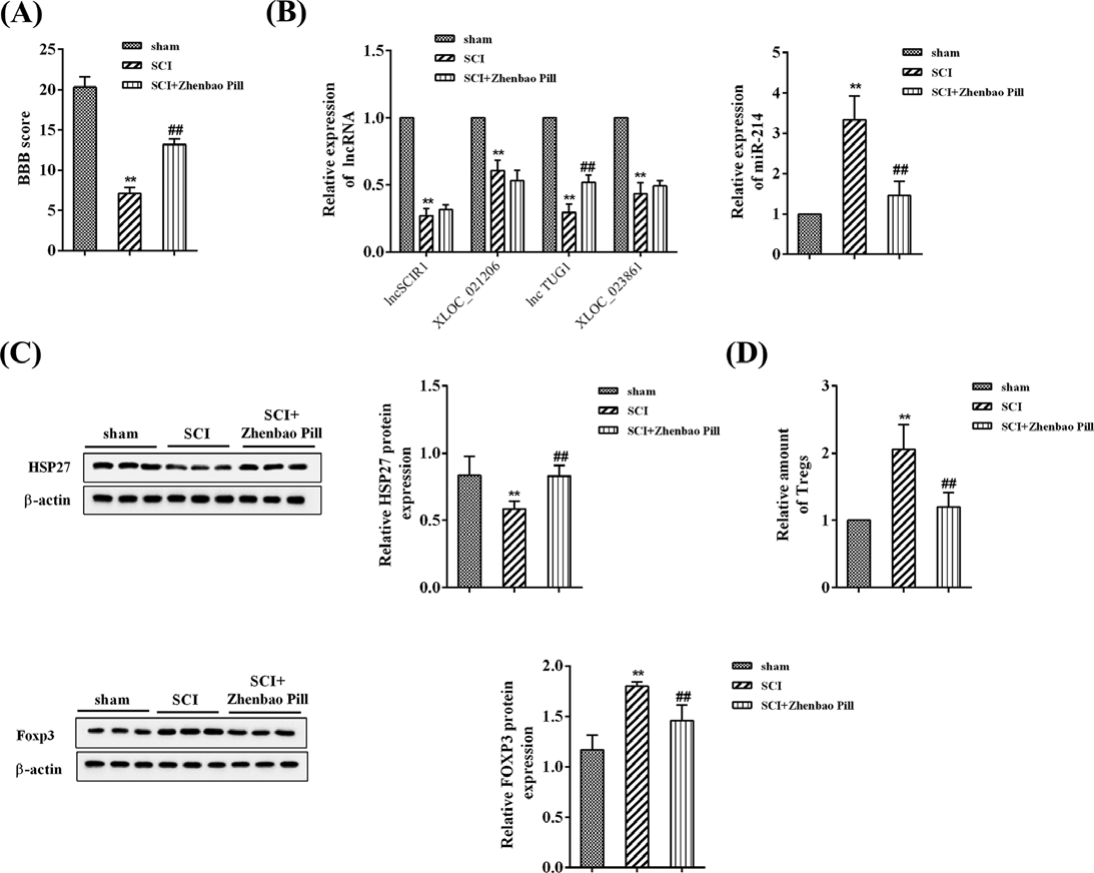
The effects of Zhenbao Pill in SCI rats Three groups were included: sham group, SCI group, and SCI + Zhenbao Pill group. (**A**) BBB score for assessing neurological function in rats. (**B**) Expression levels of lncRNA SCIR1, XLOC_021206, lncRNA TUG1, XLOC_02386, and *miR-214* were determined by qRT-PCR. (**C**) Expression levels of HSP27 were determined by Western blotting. (**D**) Relative amount of Treg cells was examined with flow cytometry analysis, and Foxp3 expression was determined by Western blotting. **P<0.01 compared with sham group; ^##^*P*<0.01 compared with SCI group.

Compared with sham group, the BBB score was significantly lower in SCI group, which was dramatically reversed by Zhenbao Pill (Figure 1A). SCI rats had lower expression of SCIR1, RGD1559747, TUG1, and XLOC_001451 than sham rats. However, only the expression of TUG1 was significantly increased after Zhenbao Pill treatment. Zhenbao Pill down-regulated the high expression of *miR-214* induced by SCI (Figure 1B). Besides, Zhenbao Pill up-regulated the decrease in HSP27 expression caused by SCI (Figure 1C). Zhenbao Pill also resulted in decreases in the proportion of Treg cells and Foxp3, Treg cell-specific marker, levels increased by SCI (Figure 1D).

### The effects of Zhenbao Pill in *in vitro* cultured CD4^+^ T cells

**Figure 2:**
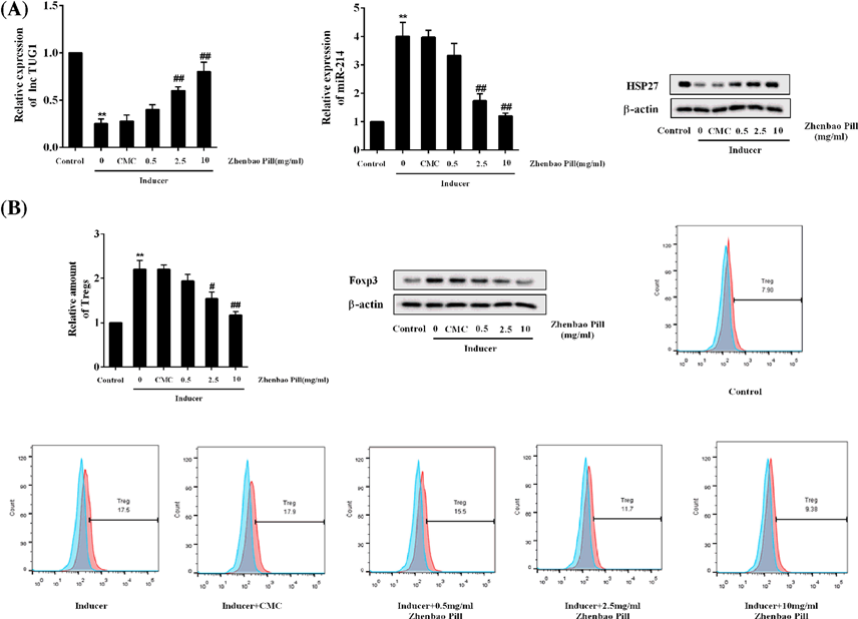
The effects of Zhenbao Pill in *in vitro* cultured CD4+ T cells CD4^+^ T cells were isolated and purified from normal rats and subjected to differentiation induction. Six groups were included: control group, inducer group, inducer + CMC group, inducer + Zhenbao Pill (0.5 mg/ml) group, inducer + Zhenbao Pill (2.5 mg/ml) group, and inducer + Zhenbao Pill (10 mg/ml) group. (**A**) Expression levels of lncRNA TUG1, *miR-214*, and HSP27 protein. (**B**) The relative amount of Treg cells, and the Foxp3 protein levels. **P<0.01 compared with control group; ^#^*P*<0.05, ^##^*P*<0.01 compared with inducer + CMC group.

Similar results were observed in *in vitro* cultured CD4^+^ T cells, Zhenbao Pill promoted the expression of TUG1 and HSP27 protein, and inhibited the expression of *miR-214* in a concentration-dependent manner (Figure 2A). Moreover, Zhenbao Pill reduced the proportion of Treg cells and Foxp3 protein levels in a concentration-dependent manner (Figure 2B).

### Interference with TUG1 abolishes the inhibitory effect of Zhenbao Pill on Treg cells

**Figure 3:**
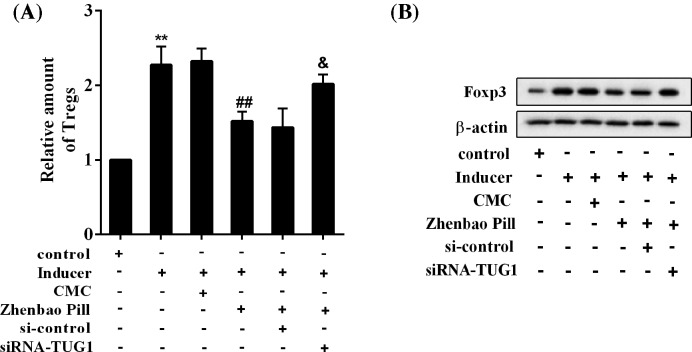
Interference with TUG1 abolished the inhibitory effect of Zhenbao Pill on Treg cells CD4^+^ T cells were isolated and purified from normal rats and subjected to differentiation induction. Six groups were included: control group, inducer group, inducer + CMC group, inducer + Zhenbao Pill group, inducer + Zhenbao Pill + si-control group, and inducer + Zhenbao Pill + siRNA-TUG1 group. The concentration of Zhenbao Pill was 2.5 mg/ml. (**A**) The relative amount of Treg cells. (**B**) Protein expression levels of Foxp3. **P<0.01 compared with control group; ^##^*P*<0.01 compared with inducer + CMC group; ^&^*P*<0.05 compared with inducer + Zhenbao Pill + si-control group.

Zhenbao Pill reduced the proportion of Treg cells, which was canceled by knockdown of TUG1 (Figure 3A). Zhenbao Pill inhibited the Foxp3 protein levels, and knockdown of TUG1 also reversed this effect of Zhenbao Pill (Figure 3B).

### TUG1 targets *miR-214*

**Figure 4:**
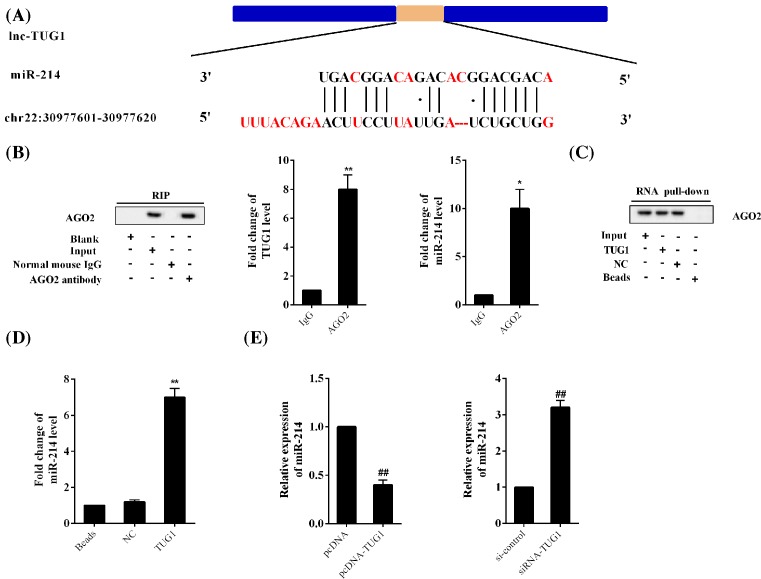
TUG1 targetted *miR-214* The following experiments were performed in 293T cells. (**A**) Software predicted the *miR-214*-binding site on TUG1. (**B**) RIP: AGO2 antibody for RNA immunoprecipitation (RIP). AGO2 was detected using IP-Western blotting, and TUG1 and *miR-214* were detected using qRT-PCR. (**C**) RNA pull-down: Western blotting was used to detect AGO2 in the TUG1 pulldown complex. NC was the TUG1 control group. (**D**) Expression of *miR-214* in the TUG1 pulldown complex. (**E**) Lentiviral transfection of rat CD4^+^ T cells was performed to overexpress or knockdown TUG1, and qRT-PCR was used to detect *miR-214* expression. *P<0.05, **P<0.01 compared with IgG or NC; ^##^*P*<0.01 compared with pcDNA or si-control.

The *miR-214*-binding site on TUG1 was predicted using bioinformatics software (Figure 4A). TUG1 and *miR-214* accumulated in AGO2. Compared with IgG, AGO2 antibody detected a large amount of TUG1 and *miR-214* (Figure 4B). AGO2 was found in the TUG1 or NC pulldown complex (Figure 4C). *miR-214* was enriched in the TUG1 pulldown complex, with only a slight increase in the NC pulldown complex (Figure 4D). Further, overexpression of TUG1 inhibited the expression of *miR-214*, and interfering TUG1 promoted the expression of *miR-214* (Figure 4E).

### TUG1 affects Treg cell differentiation through *miR-214*

**Figure 5:**
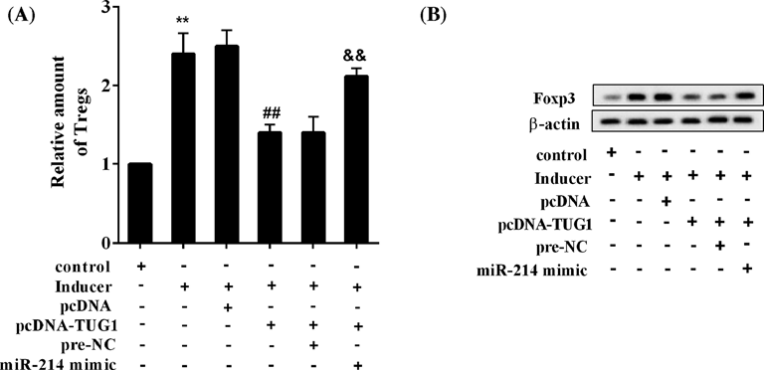
TUG1 affected Treg cell differentiation through *miR-214* CD4^+^ T cells were isolated and purified from normal rats and subjected to differentiation induction. Eight groups were included: control group, inducer group, inducer + pcDNA group, inducer + pcDNA-TUG1, inducer + pcDNA-TUG1 + pre-NC, and inducer + pcDNA-TUG1 + *miR-214* mimic group. (**A**) The relative amount of Treg cells. (**B**) Protein expression levels of Foxp3. **P<0.01 compared with control; ^##^*P*<0.01 compared with inducer + pcDNA; ^&&^*P*<0.01 compared with inducer + pcDNA-TUG1 + pre-NC.

The results showed that the inducer up-regulated the proportion of Treg cells, overexpression of TUG1 reversed the effect of inducer, and *miR-214* mimic abolished the effect of TUG1 overexpression (Figure 5A). Moreover, the inducer up-regulated Foxp3 protein expression, overexpression of TUG1 reversed the effect of inducer, and *miR-214* mimic canceled the role of TUG1 overexpression (Figure 5B).

### Zhenbao Pill modulates HSP27 expression and affects Treg cell differentiation through TUG1/*miR-214*

**Figure 6:**
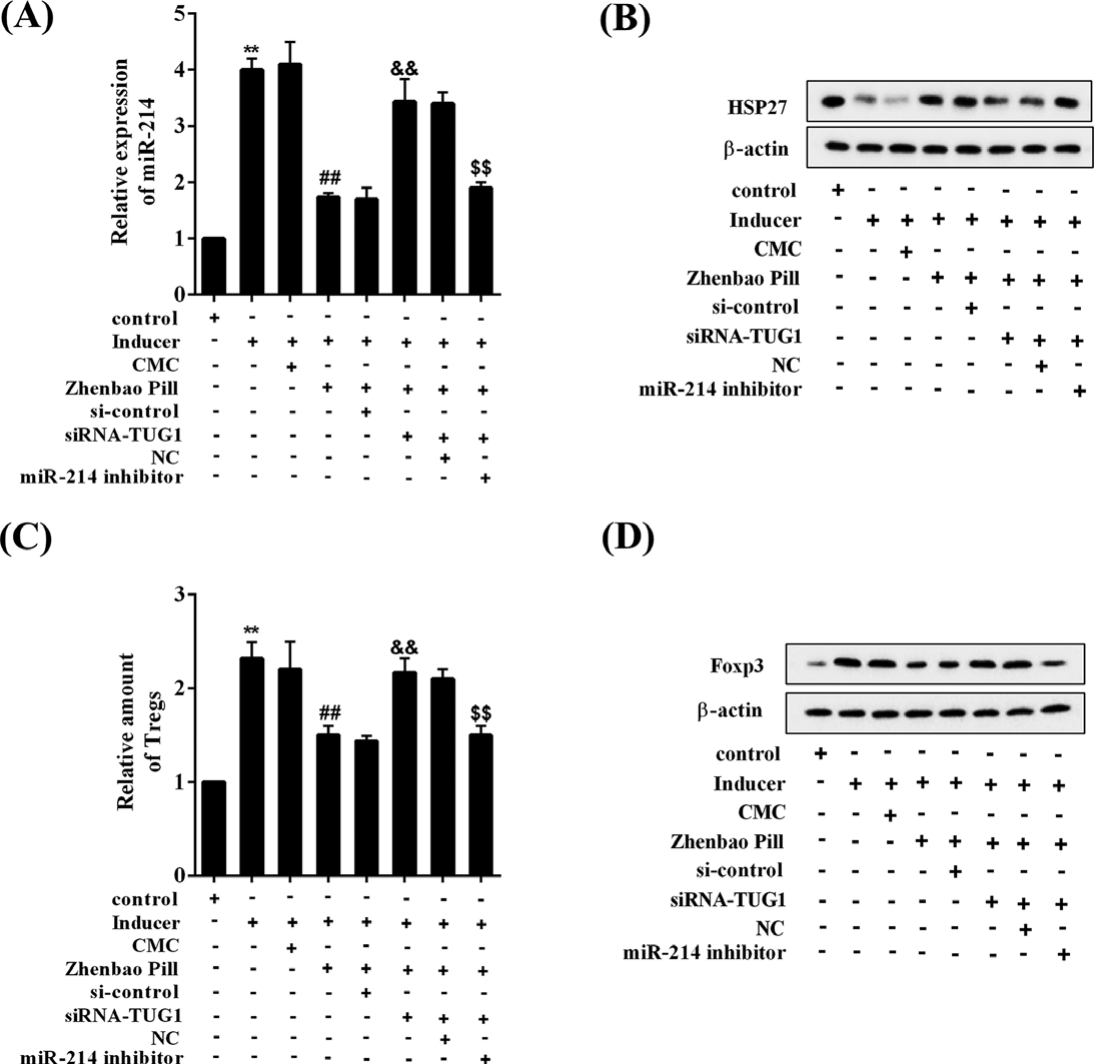
Zhenbao Pill modulated HSP27 expression and affected Treg cell differentiation through TUG1/*miR-214* CD4^+^ T cells were isolated and purified from normal rats and subjected to differentiation induction. Eight groups were included: control group, inducer group, inducer + CMC group, inducer + Zhenbao Pill group, inducer + Zhenbao Pill + si-control group, inducer + Zhenbao Pill + siRNA-TUG1 group, inducer + Zhenbao Pill + siRNA-TUG1 + NC group, and inducer + Zhenbao Pill + siRNA-TUG1 + *miR-214* inhibitor group. The concentration of Zhenbao Pill was 2.5 mg/ml. (**A**) Expression levels of *miR-214*. (**B**) Protein expression levels of HSP27. (**C**) The relative amount of Treg cells. (**D**) Protein expression levels of Foxp3. **P<0.01 compared with control group; ^##^*P*<0.01 compared with inducer + CMC group; ^&&^*P*<0.01 compared with inducer + Zhenbao Pill + si-control group; ^$$^*P*<0.01 compared with inducer + Zhenbao Pill + siRNA-TUG1 + NC group.

As shown in Figure 6A, knockdown of TUG1 reversed the low expression of *miR-214* induced by Zhenbao Pill, and *miR-214* inhibitor abolished the effect of TUG1 knockdown. Knockdown of TUG1 reversed the high protein expression of HSP27 induced by Zhenbao Pill, and *miR-214* inhibitor canceled the TUG1 knockdown effect (Figure 6B). Knockdown of TUG1 reversed the inhibition of Treg cell proportion by Zhenbao Pill, and *miR-214* inhibitor abolished the effect of TUG1 knockdown (Figure 6C). Besides, knockdown of TUG1 reversed the inhibitory effect of Zhenbao Pill on Foxp3 protein expression, and *miR-214* inhibitor eliminated the effect of TUG1 knockdown (Figure 6D).

### *miR-214*/HSP27 affects Treg cell differentiation

**Figure 7:**
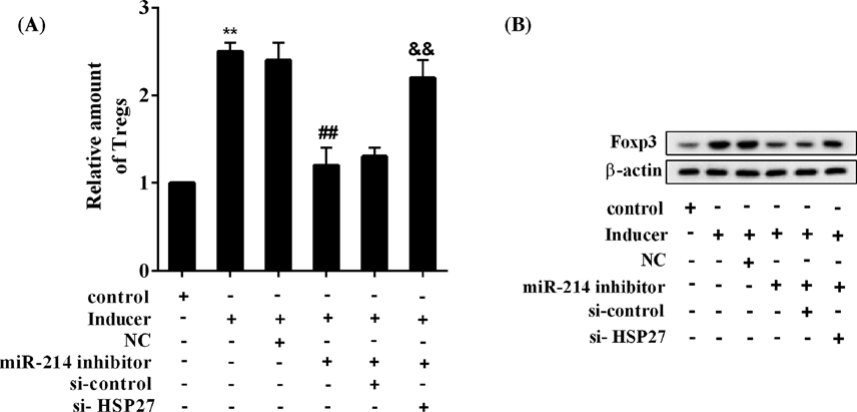
*miR-214*/HSP27 affected Treg cell differentiation CD4^+^ T cells were isolated and purified from normal rats and subjected to differentiation induction. Six groups were included: control group, inducer group, inducer + NC group, inducer + *miR-214* inhibitor group, inducer + *miR-214* inhibitor + si-control group, and inducer + *miR-214* inhibitor + si-HSP27 group. (**A**) The relative amount of Treg cells. (**B**) Protein expression levels of Foxp3. **P<0.01 compared with control group; ^##^*P*<0.01, inducer + NC group; ^&&^*P*<0.01, inducer + *miR-214* inhibitor + si-control group.

The results showed that inducer up-regulated the proportion of Treg cells, *miR-214* inhibitor reversed the effect of inducer, and knockdown of HSP27 abolished the *miR-214* inhibitor effect (Figure 7A). In addition, the inducer up-regulated Foxp3 protein expression, *miR-214* inhibitor reversed the effect of inducer, and knockdown of HSP27 eliminated the effect of *miR-214* inhibitor (Figure 7B).

### Zhenbao Pill affects Treg cell differentiation by *miR-214*/HSP27

**Figure 8:**
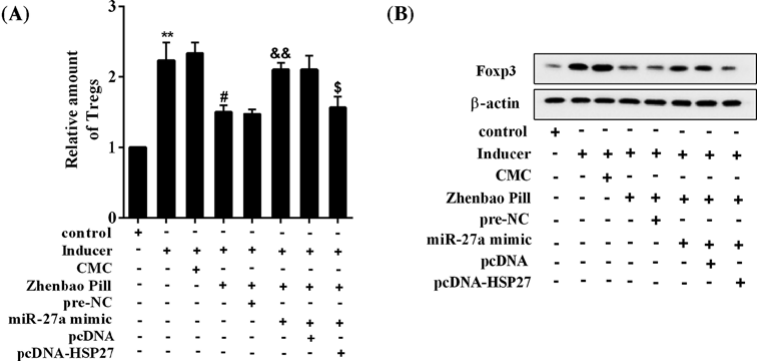
Zhenbao Pill affected Treg cell differentiation by *miR-214*/HSP27 CD4^+^ T cells were isolated and purified from normal rats and subjected to differentiation induction. Eight groups were included: control group, inducer group, inducer + CMC group, inducer + Zhenbao Pill group, inducer + Zhenbao Pill + pre-NC group, inducer + Zhenbao Pill + *miR-214* mimic group, inducer + Zhenbao Pill + *miR-214* mimic + pcDNA group, and inducer + Zhenbao Pill + *miR-214* mimic + pcDNA-HSP27 group. The concentration of Zhenbao Pill was 2.5 mg/ml. (**A**) The relative amount of Treg cells. (**B**) Protein expression levels of Foxp3. **P<0.01 compared with control group; ^#^*P*<0.05, inducer + CMC group; ^&&^*P*<0.01, inducer + Zhenbao Pill + pre-NC group; ^$^*P*<0.05 compared with inducer + Zhenbao Pill + *miR-214* mimic + pcDNA group.

As shown in Figure 8A, the *miR-214* mimic reversed the inhibition of Treg cell proportion by Zhenbao Pill, and this *miR-214* mimic effect was abolished by overexpression of HSP27 (Figure 8A). Besides, the *miR-214* mimic reversed the inhibitory effect of Zhenbao Pill on Foxp3 protein expression, which was abolished by the overexpression of HSP27 (Figure 8B).

### Zhenbao Pill relieves rat SCI with TUG1

**Figure 9:**
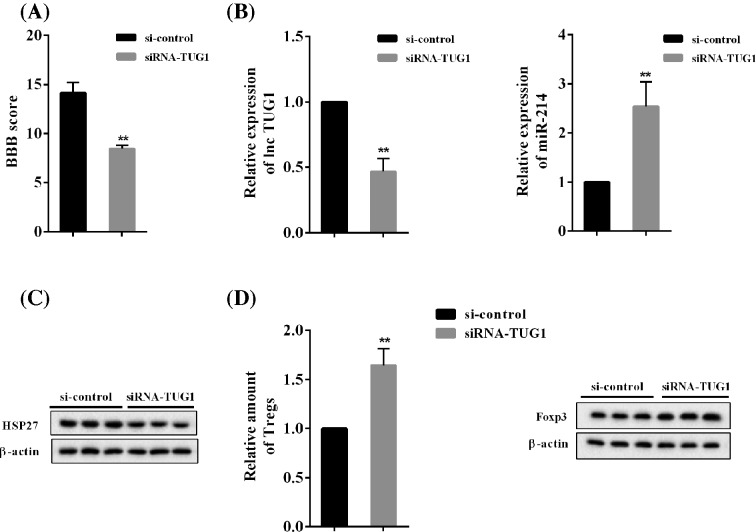
Zhenbao Pill relieved rat SCI with TUG1 Two groups were included: Zhenbao Pill + si-control group and Zhenbao Pill + siRNA-TUG1 group. (**A**) BBB score for assessing neurological function in rats. (**B**) CD4^+^ T cells were isolated and purified, and TUG1 and *miR-214* expression levels were determined. (**C**) Protein expression levels of HSP27. (**D**) The relative amount of Treg cells, and protein expression levels of Foxp3. **P<0.01 compared with Zhenbao Pill + si-control group.

Compared with Zhenbao Pill + si-control group, the BBB score was significantly decreased in Zhenbao Pill + siRNA-TUG1 group (Figure 9A). It was found that knockdown of TUG1 inhibited the expression of TUG1 and promoted the expression of *miR-214* (Figure 9B). Besides, knockdown of TUG1 inhibited the protein expression of HSP27 (Figure 9C). Further, knockdown of TUG1 up-regulated the proportion of Treg cells, and Foxp3 protein levels (Figure 9D).

## Discussion

In the present study, we found that Zhenbao Pill promoted the expression of TUG1 and HSP27 protein, and reduced the expression of *miR-214* and Foxp3 protein as well as the proportion of Treg cells in a concentration-dependent manner in both SCI rats and *in vitro* cultured CD4^+^ T cells. Importantly, Zhenbao Pill relieved rat SCI with TUG1. We finally concluded that Zhenbao Pill regulated the TUG1/*miR-214*/HSP27 signaling pathway to reduce Treg cell proportion and thus relieve acute SCI.

SCI is one of the weakest pathologies that seriously affect the quality of life of patients. It has been reported that approximately 2.5 million people suffered from SCI, with over 130000 new cases found each year [24,25]. SCI could be divided into primary SCI and secondary SCI. Primary SCI is caused by violence against the spine. Secondary SCI results from multiple complex mechanisms, such as neuro-inflammation, oxidative stress, neuronal injury etc., further damaging the neurological and psychological functions of patients [26]. As the primary SCI is irreversible, treatment of the secondary SCI has become the focus of clinical practice [27]. As reported, Zhenbao Pill could repair the damaged neurones, promote microcirculation, and remove oxygen free radicals [8,9], suggesting the potential application of Zhenbao Pill in treating SCI patients. Indeed, Zhenbao Pill has been shown to protect SCI rats from further damage [10,11], with improved BBB score. Consistent with this finding, in the present study, we also found that the BBB score was significantly lower in SCI group compared with sham group, which was dramatically reversed by Zhenbao Pill.

Following SCI, lots of immune cells are activated and gathered into the damaged spinal area, including Treg cells. In an ASCI model, decrease in Treg cells were found to enhance the functional recovery following SCI, indicating that alleviated systemic immunosuppression could promote SCI repair [12]. Our previous study found that Zhenbao Pill inhibited the Treg lymphocyte amount by inhibiting the *miR-214* induced expression of HSP27 during the repair of SCI [11]. This finding was also further confirmed by the present study: *miR-214* mimic reversed the inhibition of Treg cell proportion and Foxp3 protein expression by Zhenbao Pill, which was abolished by the overexpression of HSP27. Besides, recently, we also found that Zhenbao Pill protected against ASCI through regulating the miR-146a-5p/G-protein-coupled receptor 17 (GPR17) signaling pathway [10], indicating that miRNAs contribute a lot to the treatment of SCI by Zhenbao Pill.

We next investigated the underlying mechanism of how *miR-214* is regulated in SCI. LncRNA is a class of non-coding RNA that has been implicated in multiple physiopathological processes, including SCI [28,29]. In a contusion SCI mouse model, the expression of numerous lncRNAs was found to be changed using microarray [30], indicating the potential role of lncRNAs in the pathogenesis of SCI. For example, Wang et al. [31] found that lncSCIR1 was down-regulated following SCI, and knockdown of lncSCIR1 could promote the astrocyte proliferation and migration *in vitro*, suggesting that lncSCIR1 might participate in the pathogenesis of SCI. TUG1 could promote cell proliferation, and is up-regulated in many tumor cells, including glioma, small cell lung cancer, and hepatocellular carcinoma [13,14,32]. Further, as reported, it can act as a sponge for many miRNAs, including miR-26a, and miR-145 [33,34]. Studies have demonstrated that TUG1 was expressed in PBMCs and T lymphocytes [15,16]. Software (LncBase Predicted v.2) predicted the binding between TUG1 and *miR-214*. Therefore, in the present study, we investigated the role of TUG1 in SCI. The results showed that interference with TUG1 abolished the inhibitory effect of Zhenbao Pill on Treg cells. TUG1 could serve as an *miR-214* sponge, and TUG1 affected Treg cell differentiation through *miR-214*. Further, Zhenbao Pill reduced the proportion of Treg cells by regulating the expression of HSP27 through TUG1/*miR-214* and thus improved ASCI. Compared with previous study [11], our study further elucidated the underlying molecular mechanism of how Zhenbao Pill reduced the proportion of Treg cells and improved ASCI. However, the TUG1/*miR-214*/HSP27 signaling pathway should be further confirmed *in vivo* in the future studies.

## Conclusion

In conclusion, Zhenbao Pill regulated the TUG1/*miR-214*/HSP27 signaling pathway to reduce Treg cell proportion and thus relieve acute SCI, providing the theoretical basis for the clinical use of Zhenbao Pill in treating SCI patients.

## Abbreviations

AGO2: argonaute 2
ASCI: acute spinal cord injury
BBB: Basso, Beattie, and Bresnahan
Foxp3: forkhead box protein p3
HSP27: heat shock protein 27
lncRNA: long non-coding RNA
NC: negative control
PBMC: peripheral blood mononuclear cell
SCI: spinal cord injury
SD: standard deviation
TGF-β: transforming growth factor-β
TUG1: taurine up-regulated gene 1
